# Community-Based Family Health History Education: The Role of State Health Agencies in Engaging Medically Underserved Populations in Understanding Genomics and Risk of Chronic Disease

**DOI:** 10.3390/healthcare3040995

**Published:** 2015-10-21

**Authors:** Laura Senier, Michael Shields, Rachael Lee, Lauren Nicoll, Danielle Falzon, Elyssa Wiecek

**Affiliations:** 1 Department of Sociology and Anthropology, Northeastern University, 360 Huntington Ave Boston, MA 02115, USA; E-Mails: m.shields@neu.edu (M.S.); r.lee@neu.edu (R.L.); l.nicoll@neu.edu (L.N.); d.falzon@neu.edu (D.F.); 2 Department of Health Sciences, Northeastern University, 360 Huntington Ave Boston, MA 02115, USA; 3 School of Pharmacy, Northeastern University, 140 Fenway, 360 Huntington Ave Boston, MA 02115, USA; E-Mail: wiecek.el@husky.neu.edu

**Keywords:** family history, genomics, chronic disease prevention, state health agencies, public health, program evaluation

## Abstract

Although family health history (FHH) collection has been recognized as an influential method for assessing a person’s risk of chronic disease, studies have shown that people who are low-income, from racial and ethnic minorities, and poorly educated are less likely to collect their FHH or share it with a medical professional. Programs to raise public awareness about the importance of FHH have conventionally targeted patients in primary care clinics or in the general community, but few efforts have been made to coordinate educational efforts across settings. This paper describes a project by the Connecticut Department of Public Health’s Genomics Office to disseminate training materials about FHH as broadly as possible, by engaging partners in multiple settings: a local health department, a community health center, and two advocacy organizations that serve minority and immigrant populations. We used a mixed methods program evaluation to examine the efficacy of the FHH program and to assess barriers in integrating it into the groups’ regular programming. Our findings highlight how a state health department can promote FHH education among underserved communities.

## 1. Background

Family health history (FHH) collection has been recognized as a simple yet informative tool for predicting an individual’s risk of developing chronic diseases such as cancer, diabetes, or heart disease. It has been touted as a “tried-and-true” method for cheaply assessing a patient’s susceptibility to chronic diseases, based on an assessment of genetic, environmental, and social predictors [1]. It provides both patients and providers with knowledge to personalize disease prevention plans [1,2,3,4].

Its use, however, remains limited among certain demographic groups. Individuals of lower socioeconomic status and certain racial or ethnic minorities have consistently reported lower levels of collecting FHH information and sharing it with a medical provider [5,6,7,8]. The underutilization of this risk assessment tool reflects many structural and cultural barriers. The American healthcare workforce does not reflect the diversity in the general population and many providers lack the cultural competency to communicate disease prevention and management strategies to non-white and low-income patients [9,10,11,12]. Many nonwhite communities are also suspicious of and reluctant to engage the healthcare system because of a history of mistreatment and discrimination [5,13]. As a result, underserved communities face many barriers in accessing health services or health educational programming [9,10,14]. Because these underserved groups suffer disproportionally from higher incidence of chronic disease, more concerted efforts are needed to teach them about risk assessment and disease prevention [15]. Community-based FHH educational interventions are a promising method for advancing this goal.

In an effort to promote broader utilization of FHH collection and address disparities in its use, two types of educational programs have been developed: teaching primary care providers (PCPs) to collect and interpret FHH (to quickly identify patients who might need follow-up screening or diagnostic assessment), or teaching the public about the importance of collecting one’s FHH and sharing it with a healthcare provider.

Multiple FHH educational interventions have been implemented in primary care settings [16,17]. However, barriers of insurance coverage, medical cost, and spatial distribution limit PCPs’ interaction with disadvantaged populations and make these FHH interventions largely ineffective at reaching underserved groups [12,18]. Moreover, many PCPs have found collection of FHH data burdensome in the primary care setting [19,20]. Proper pedigree construction is complicated and time consuming [4,19,21,22] and many PCPs have reported low to moderate levels of confidence in their ability to construct pedigrees and use that information to personalize chronic disease prevention and treatment [23,24]. Williams *et al.*’s analysis of PCP’s collection of FHH revealed that time constraints and incompleteness of patients’ FHH information caused some PCPs to abandon FHH collection, even though they believed it useful for disease prevention [21].

In order to alleviate the time consuming task of FHH collection and provide PCPs with FHH information, many interventions have encouraged patients to collect their own FHH information so that they can share it with a PCP. Several online assessment tools, such as the popular *MeTree* risk assessment software or the U.S. Surgeon General’s *My Family Health Portrait* online toolkit, allow patients to collect their FHH and generate a printout to share with a PCP [19,25,26]. Yet these FHH interventions do not address the barriers of mistrust and miscommunication experienced by disadvantaged groups [5,10,17]. The online tools also are inaccessible to patients without access to computers or the Internet [21].

These disappointing results have led to the development of educational programs that directly address the public. For example, the U.S. Surgeon General has designated Thanksgiving Day as *National* *Family History Day*; this and other broad-based public awareness campaigns encourage people to talk with their relatives over the holidays about their FHH [27]. More direct educational programs have been offered in small group settings. Such projects frequently entail partnerships between academic researchers and local community organizations [5,6,7,28,29,30]. Few community-level interventions, however, have targeted FHH educational programing specifically for underserved communities. A notable exception was a campaign initiated in 2008 by the Genetic Alliance, a national patient advocacy organization. The Genetic Alliance convened an expert group of medical professionals, oral historians, patient advocates, and community representatives to create a customizable and culturally accessible FHH toolkit [31].The result was the *Does It Run In The Family?* FHH toolkit, which the Genetic Alliance promoted as tool “produced by the community, for the community [18,31,32].” The Genetic Alliance partnered with 10 community groups, who were advised to customize the FHH toolkit with community member narratives and then offer FHH educational programs alongside their existing programs. The Genetic Alliance’s message to these organizations was that, “Family health history should not be a stand-alone intervention. Integration is key for sustainability [18].” The Genetic Alliance found that the community groups enjoyed customizing the educational materials to better inform constituents about chronic disease risks, and that participants reported greater interest in and willingness to share their FHH with their relatives and providers. The Genetic Alliance recommended that future interventions follow this two-pronged approach of customization and integration as a successful method for promoting FHH collection in underserved communities. The Genetic Alliance’s evaluation of this effort, however, focused primarily on the usability of the toolkit and did not assess whether the groups continued to offer the intervention after the initial project period [18,31,33,34].

State health agencies can be important champions for disseminating information about novel health promotion practices, because they have such strong partnerships with many different sectors of the community. Since 2008, the Connecticut Department of Public Health’s (CT-DPH) Genomics Office has promoted FHH education to inform residents about the role of heredity in the development of chronic disease [35]. In 2008, the CT-DPH Genomics Office created the *Family Health History & Chronic* *Disease Workbook* to increase awareness of the topic and promote FHH collection in Connecticut [36]. The workbook is based on the U.S. Surgeon General’s *My Family Health Portrait* toolkit and contains general information on the topic of FHH, its relation to a person’s risk of developing chronic disease, a blank pedigree for participants to complete, and instructions on sharing the information with relatives and healthcare providers. The CT-DPH Genomics Office also created disease-specific insert cards for the workbook; these cards highlight certain heritable chronic conditions and their symptoms [36,37]. The CT-DPH Genomics Office has disseminated the workbook to healthcare providers, health systems, and patient advocacy organizations, and offered it at health fairs and conferences across the state [36].

In an effort to more directly engage underserved populations about the importance of knowing one’s FHH, the CT-DPH Genomics Office partnered with researchers at Northeastern University on the *Connecticut Community Family Health History Project* pilot study. The goal of this pilot study was to provide training in FHH collection to medically underserved communities in Connecticut. Previous assessments conducted by the CT-DPH Genomics Office using the *Behavioral Risk Factor Surveillance* *System* (BRFSS) have shown wide disparities in the incidence and prevalence of chronic conditions such as heart disease, stroke, diabetes, and cancer, and that low-income and minority patients are least likely to report collecting FHH information or sharing it with a medical provider [36]. In fact, although Connecticut ranks favorably in many health indicators (e.g., health insurance coverage, infectious disease incidence, smoking and obesity prevalence), it experiences striking health disparities [38,39,40,41]. For example, the mortality rate for heart disease is 1.2 times higher among black or African American Connecticut residents than white residents, while the mortality rate for diabetes is 1.5 times higher among Latino/Hispanic Connecticut residents than white residents [40]. FHH collection and promotion may be an effective method for reaching these at-risk populations in need of invaluable medical information.

The CT-DPH Genomics Office believed the best means of reaching these groups with a FHH educational program would be to network with community-based health organizations. However, unlike previous research, this pilot study focuses on how a state health agency can best identify and prepare community-based organizations to integrate FHH programming into their ongoing programming, rather than simply assessing the utility of the FHH toolkit itself. We report how four community-based organizations customized Connecticut’s FHH educational program materials to meet the needs of their constituents; evaluate their effectiveness at engaging underserved communities in the topic of FHH; and assess the likelihood of sustaining FHH collection in the group’s future programming.

## 2. Methods

In the autumn of 2014, the CT-DPH Genomics Office’s program coordinator purposively identified four community-based organizations that reached low-income or ethnic minority populations; she invited them to develop proposals using the existing FHH curricular material, and offered $1000 mini-grants to implement a FHH educational intervention for their patients or clients. These organizations included a local health department, a federally qualified health center, and two nonprofit advocacy groups that work with ethnic minority and immigrant populations. Each community organization received copies of the CT-DPH Genomics Office’s *Family Health History & Chronic Disease Workbook* (CT-DPH FHH workbook) and an orientation on FHH collection methods from the CT-DPH Genomics Office coordinator. They each proposed FHH interventions that were customized to meet the needs of their target populations. The CT-DPH Genomics Office coordinator reviewed and approved the proposals before implementation began. Table 1 describes the four organizations, the populations they serve, and how they incorporated the FHH intervention into their existing programming. It should be noted there may be some overlap between the four community-based organizations. Some residents may have been reached by multiple organizations’ FHH interventions.

Each organization proposed a FHH intervention tailored to the audience it serves. The Northeast District Department of Health (NDDH) proposed a public awareness campaign to inform residents in Connecticut’s northeast corner about FHH [42] The NDDH proposed producing a public service announcement with a local radio station to advertise the U.S. Surgeon General’s designation of Thanksgiving Day as *National Family History Day* [27]. They also proposed promoting the topic at a winter holiday parade in Putnam, Connecticut. The local radio station is a nonrated AM station that reaches approximately 105,000 individuals in both Connecticut and Massachusetts from its central broadcasting location in Putnam, Connecticut [43]. The Holiday Dazzle Light Parade has drawn approximately 100,000 people for the past 13 years [44].

The Community Health Center, Inc. (CHC, Middletown, CT, USA) is a federally qualified health center based in Middletown, Connecticut; clinical professionals offer affordable healthcare at the main clinic and at 13 satellite clinics statewide. In addition to providing acute and preventive clinical services, the CHC has approximately 15 AmeriCorps volunteers each year who are trained to offer small-group educational programming on topics such as chronic disease risk, oral hygiene, smoking cessation, and nutrition and physical fitness [45]. According to the CHC, the AmeriCorps volunteers organize approximately five outreach events each year, each of which typically engages approximately 250 families [46]. The CHC proposed training the AmeriCorps volunteers to run the intervention at three previously scheduled community outreach events and planned to disseminate both the CT-DPH FHH workbook and the Genetic Alliance’s *Does It Run In The Family?* FHH toolkit.

The Hispanic Health Council (HHC) proposed integrating FHH education into its *Comadrona*/Healthy Start prenatal case management program. The *Comadrona*/Healthy Start Program is subsidized by federal, state, and private funds and is staffed by community health workers and case managers who provide counseling and aid to approximately 255 expectant mothers annually, in on-site health consultations or at-home visits [47,48]. During these health consultations, the community health workers provide information about healthy pregnancies, assist with enrollment in health insurance or government programs, act as midwives, and help participants with daily errands and appointments [47]. Although it offers comprehensive wrap-around care, its reach is somewhat limited, because it caters solely to expectant mothers in a community of 141,000 Latino/Hispanic individuals in the greater Hartford area[49] (See Table 1). The HHC proposed incorporating FHH training into routine health consultations with 30 newly registered expectant mothers.

The Khmer Health Advocates (KHA) proposed integrating the FHH intervention into an ongoing oral history initiative to document family histories of trauma resulting from the Cambodian genocide in the late 1970s. In 1992, they created a bilingual *Cambodian Survivor Health Assessment* tool to assist community members in collecting narratives of personal and family trauma of the genocide and migration, and how it has impacted their mental health and well-being [50]. KHA staff members have used the assessment tool in health consultations at the organization’s clinic. The KHA planned to add CT-DPH Genomics Office’s FHH module to further emphasize FHH’s relation to chronic diseases. KHA staff also wished to establish a relationship with second-generation youth in the community and proposed training 10 youth outreach educators to collect their own FHH and educate older relatives about how FHH relates to chronic disease.

**Table 1:** Family health history (FHH) interventions completed by Connecticut community-based health groups.

Community Group	Program Type	Location Served	Population Served*	FHH Intervention
Northeast District Department of Health (NDDH)	A local health department providing public health information and services [42].	The “Northeast Corner” of Connecticut consisting of 438 square miles in Windham County and one town in Tolland County (approximately 2.4% of Connecticut’s total population) [41,42].	The 85,666 residents of Connecticut’s “Northeast Corner” predominantly identify as white (89.6%) with only 8.7% of households living below the poverty line [42,51].	Conducted awareness campaign in the Northeast Corner with a radio public service announcement and disseminated handouts at a community holiday parade.
Community Health Center, Inc. (CHC)	A private non-profit primary health care system providing affordable, culturally-competent primary care services to un- and underinsured, patients [52].	Operates health centers in Bristol, Clinton, Danbury, Enfield, Groton, Meriden, Middletown, New Britain, New London, Norwalk, Old Saybrook, Stamford, and Waterbury [53].	Serves approximately 130,000 patients annually; predominantly Hispanic/Latino (47.3%) followed by white (40.2%). Approximately 20% of the patient population remains uninsured while 62% receives public coverage [52,54].	Trained AmeriCorps members to incorporate FHH education and disseminate educational materials at community events.
Hispanic Health Council (HHC)	A private non-profit health advocacy and social justice organization dedicated to improving the health and social being of Latinos and low income inner-city populations [55].	Provides culturally relevant direct health and social service programs from centrally located health center in Hartford, CT [55].	Serves approximately 141,000 Hispanic or Latino individuals in the greater Hartford area [49]. The Latino/Hispanic population within Connecticut report higher rates of poverty (37%) and higher rates of uninsured (21%) than any other racial/ethnic groups within the state [56].	Trained community health workers to include FHH questions in the *Comadrona*/ Healthy Start prenatal case management program.
Khmer Health Advocates (KHA)	A private non-profit health advocacy organization dedicated to caring for the health of Cambodian populations and survivors of the Cambodian genocide [50].	Provides culturally relevant direct health service programs to the Cambodian populations in Connecticut and Western Massachusetts, from centrally located health center in West Hartford, CT [50].	Advocates for and serves the approximately 2,772 Cambodians in New England area [49]. Cambodian populations have reported high rates of poverty and are three times less likely to visit a physician than the general U.S. population [57,58].	Educated youth members in FHH collection, incorporated FHH discussion into regular health consultations, and conducted community outreach at Cambodian New Year celebration.

Note: * Some population overlap may exist between the four community-based organizations. Some residents may have been reached by multiple organizations’ FHH intervention.

Our evaluation of these community-level FHH interventions included both a process evaluation and an outcome evaluation. This dual evaluation framework allowed us to assess both (1) the feasibility of incorporating the FHH interventions into each organization’s regular programming and (2) the impact of the interventions on participants’ perceptions and knowledge of FHH. 

The process evaluation allowed us to examine the correspondence between the objectives of the pilot study and the components of the community organizations’ interventions [59]. It posed the following questions: (1) did the intervention promote the topic of family health history to underserved communities; (2) what methods and resources were used; (3) what barriers did they encounter in implementing their interventions as planned; and (4) what barriers, if any, do they anticipate in incorporating the FHH program into their regularly scheduled programming. To answer these questions, one member of the research team conducted on-site observations of the groups’ orientation sessions and community outreach events while two team members separately compared the final reports the organizations submitted to the original plans outlined in the proposals. These two team members wrote reports summarizing the differences and similarities between the groups’ original proposals and final reports and brought them to a research team meeting to resolve any discrepancies (there were none). We also provided each group with a summary sheet to record: the number of events they held, the number of participants at each session, the number of community educators present, and how many copies of the FHH materials they distributed.

The outcome evaluation determined whether the interventions produced a change in knowledge and attitudes among their target populations [59]. It answered the following questions: (1) did the intervention increase participants’ perceptions and knowledge of FHH as a risk factor for chronic disease; (2) did the intervention meet its objective of providing FHH information to underserved communities; (3) did the intervention address the educational and cultural needs of the target populations; and (4) will the partnering organizations continue promoting FHH collection. We provided a post-test survey to be administered after each intervention. It measured participants’ assessment of: the intervention, the staff educator, the FHH materials, their future intentions to use the material, whether or not the participant saw a medical professional in the past 12 months, and basic demographic information. We used *STATA* version 12 to analyze the survey data.

We shared findings from the participant surveys with site coordinators at all four community organizations in follow-up interviews. During these semi-structured interviews, we asked them to reflect on their experience with the pilot study, their opinions about the interventions’ effectiveness, and their intent to integrate the intervention into the organization’s regular programming. Interviews ranged from 30 to 60 minutes and were recorded and transcribed. We analyzed interview transcripts, fieldnotes, and the organizations’ reports in conjunction with quantitative survey results. Unreferenced quotations are drawn directly from interview transcripts.

## 3. Results

All four of the community groups organized and implemented community-level FHH interventions for their constituents. Overall, the message of FHH reached an estimated 101,300 individuals through all channels (public awareness campaigns in the media and at the community parade, and in small group sessions at each agency). A smaller number (N = 427) completed small-group sessions in the community agencies, and 160 individuals formally provided feedback on a post-test survey form.

### 3.1. Process Evaluation

#### 3.1.1. Intervention Formation and Resources Used

Although some of the organizations carried out their intervention as proposed, some interventions were modified, with the approval of the CT-DPH Genomics Office coordinator.

The NDDH carried out its proposed public awareness campaign as planned. Staff produced one 30-minute interview segment about FHH on a local radio station, which aired once before the Thanksgiving Holiday. The NDDH also piggybacked promotion of FHH in their parade float at the annual winter holiday parade in Putnam, Connecticut. The main theme of the float was to encourage individuals to get a flu shot for the winter season. In addition, three parade walkers marching beside the float carried signs saying, “It’s not too late to create your family health history. A single talk can influence your future.” Finally, they disseminated packets of tissues to parade watchers along the route; the packets had a sticker affixed to them saying, “It’s *snot* [sic] too late to create your family health history! Knowing your family’s health history can influence your future. Learn more at the *My Family Health Portrait* website!” The sticker also had links to the U.S. Surgeon General’s *Family Health History Initiative* website and the *My Family Health Portrait* online toolkit. Funds were used for the float’s construction and printing costs. An estimated 100,000 people attended the holiday parade.

The CHC incorporated FHH into its AmeriCorps training sessions and ran the intervention at four outreach events, with the first event acting as a pilot. These events included a community holiday gathering in Middletown (the pilot), a career fair at a homeless shelter in Norwalk, a neighborhood block party in Middletown, and a health fair held in conjunction with a 5K race sponsored by the CHC. Funds were used to provide snacks and refreshments at the events. The AmeriCorps volunteers disseminated only the CT-DPH FHH workbook and not the Genetic Alliance’s *Does It Run In The Family?* FHH toolkit, because of time constraints. Participant surveys were disseminated at the latter three events but not at the pilot event. The CHC reached approximately 300 families at these events.

The HHC trained community health workers to discuss FHH during regular *Comadrona*/Healthy Start Program health consultations. However, site coordinators were unsure whether community health workers provided CT-DPH FHH workbooks to all program participants (citing time constraints and the prioritization of other health concerns in these sessions). Nevertheless, information was provided to 75 expectant mothers, reaching 45 more women than originally proposed. The HCC also modified one question on the post-test participant survey; the question originally asking participants to assess the FHH handouts was substituted with a new question assessing participants’ knowledge of FHH as a direct result of the intervention (see Table 2).

The KHA modified their original proposal, because the Cambodian youth educators were not sufficiently fluent in Khmer to convey information about genomics and chronic disease to their non-English speaking relatives. As a result, they restricted their intervention to offering the FHH education sessions for the 20 youth educators. Participants were between the ages of 15–25, and they received both the *Cambodian Survivor Health Assessment* tool and the CT-DPH FHH workbook. In addition, the KHA discussed FHH and chronic disease in regular health consultations with 32 families at the clinic, making it the only organization to integrate the intervention directly into its clinical services; these patients received both toolkits. Finally, staff provided general information about FHH and chronic disease at a Cambodian New Year Celebration in Bristol, Connecticut. The KHA reached approximately 800 people at this event; however, participant surveys were disseminated only at the adolescent education sessions and in clinic health consultations.

**Table 2:** Descriptive statistics of participants’ perceptions resulting from the intervention (n = 152).

Survey Likert Scale Questions	Total (n = 152)	
	N	%
**It is important to know your own FHH**		
Not Sure Strongly Disagree Disagree In Between Agree Strongly Agree	2 1 3 16 83 47	1.3 0.6 2.0 10.5 54.7 30.9
**Knowing my FHH decreases my chances of developing future health problems**		
Not Sure Strongly Disagree Disagree In Between Agree Strongly Agree	4 0 12 25 83 28	2.6 0 7.9 16.5 54.6 18.4
**The person who helped me with the FHH materials was very helpful**		
Not Sure Strongly Disagree Disagree In Between Agree Strongly Agree	2 1 2 7 100 40	1.3 0.7 1.3 4.6 65.8 26.3
**I found the FHH handouts to be useful**		
Not Sure Strongly Disagree Disagree In Between Agree Strongly Agree	1* 1* 1* 17* 88* 24*	0.8* 0.8* 0.8* 12.9* 66.7* 18.2*
**I want to share my FHH with my relatives**		
Not Sure Strongly Disagree Disagree In Between Agree Strongly Agree	13 2 4 19 86 28	8.6 1.3 2.6 12.5 56.6 18.4
**I want to share my FHH with my doctor**		
Not Sure Strongly Disagree Disagree In Between Agree Strongly Agree	5 1 2 18 91 35	3.3 0.7 1.3 11.8 59.9 23.0
**I would participate in another event that discussed FHH**		
Not Sure Strongly Disagree Disagree In Between Agree Strongly Agree	16 2 3 29 75 27	10.5 1.3 2.0 19.1 49.3 17.8
**Because of what I learned today, I know something new about FHH**		
Not Sure Strongly Disagree Disagree In Between Agree Strongly Agree	0+ 0+ 1+ 0+ 18+ 1+	0+ 0+ 5.0+ 0+ 90.0+ 5.0+

Note: * The Hispanic Health Council deleted the survey question because they did not provide the handouts during the consultations. The total column only accounts for the Community Health Center, Inc. and Khmer Health Advocates participants (n = 132). + The Hispanic Health Council substituted this question. The total column only accounts for the Hispanic Health Council participants (n = 20).

#### 3.1.2. Successes and Barriers Affecting Sustainability

All four organizations expressed interest in and intent to continue offering community-level FHH education. The interviews with the site coordinators revealed two main factors that contributed to the interventions’ success.

First, site coordinators believed that FHH collection nicely complemented their established programming, which addressed topics such as heart disease, diabetes cancer, asthma, and obesity. However, only the KHA had previously utilized FHH as a component of health promotion programming, and only in relation to psychological trauma and mental health outcomes. All four site coordinators reported that they believed that adding an individualized risk assessment based on a patient’s family history to their chronic disease prevention messaging benefitted their constituents. One site coordinator viewed the intervention as a new way to engage local populations in chronic disease prevention.

“We’re always looking at new ways to prevent chronic diseases and interventions and all of that. So I think adding a new layer of this that we might not have done before just benefits our patients even more than what we already do. A doctor can provide advice on managing diseases but I think knowing your family health is new information being provided to the patient. It's almost empowering the patient to learn more about managing their health.”

The site coordinators also believed that the FHH program strengthened the relationship between staff and participants. Several coordinators mentioned that their providers and educators were enthusiastic to learn and share FHH information with the community. In fact, the HHC staff has proposed expanding FHH training into all of their future programs, not only those for expectant mothers, and have requested FHH training for all of their staff members. Another coordinator recounted how FHH collection made participants more comfortable with staff, “When somebody comes and says, ‘Tell me about your family.’ How many people do that in your life? It kind of says, ‘I’m interested in you.’ People usually react pretty good to that.”

A second factor that contributed to the interventions’ success is that the CT-DPH Genomics Office coordinator allowed the groups to propose something that fit their organizations’ established programming. For example, staff at the NDDH said that in their prior experience, they have found public awareness campaigns to be an effective method for introducing new topics to communities in their district. Administrators at the CHC said that they have found the HealthCorps Program’s AmeriCorps volunteers are an effective outreach staff for teaching low-income patients about preventative care. The HHC and the KHA also stated that including FHH in their health consultations provides a new way to discuss healthy habits and prevention strategies with participants. One site coordinator remarked that this flexibility was refreshing in comparison with more proscribed interventions, which typically falter over time, “[Integration is] less stressful too for whoever is running the project. I know for us, we were like, ‘Well, we can incorporate it any way we want, this is great!’”

Site coordinators also identified three challenges that may prevent them from routinely offering the FHH program, however.

First, while coordinators and staff were comfortable in providing general messages about FHH and chronic disease prevention, many feared that genetics and genomics may be too novel or complex for their participants or clients or some of their lay health educators. Most of the coordinators believed the intervention was a general first step for introducing the topic of FHH, but that more formative education would be needed to properly prepare underserved communities to understand the implications of FHH personalized health promotion initiatives that might involve genetic testing. One coordinator believed that this placed a burden on the community health organizations to further assist underserved community members in navigating the risk assessment results:

“It is so important that something this complex, with so many implications all the way through, gets explained really, really clearly. The way we did it was a superficial toss, it was: ‘Here’s some information.’ But if people are really going to use it…and then bring it to their doctor, they need [a community health advocate] holding their hand.”

To overcome this barrier, coordinators incorporated education about FHH collection into their chronic disease prevention while avoiding any discussion of genetic testing. Finally, on a related note, one site coordinator believed that using individual genetic risk to personalize health promotion strategies is too radically different from the type of health promotion strategies they typically discuss.

“There’s a big difference between genetic health history and family health history, if you ask me. Cause genetic means you’re actually making an individual specific assessment of risk and family health history is just the general risk factors that are observed in families. The standard [chronic disease] messaging are simple messages.”

Health promotion initiatives in these communities typically revolve around general messages about behavior or lifestyle modification (e.g., smoking cessation, nutrition and physical activity) that are applicable to all patients. Some site coordinators believed that using FHH to tailor a message about behavioral or lifestyle interventions would be too complex for some audiences or for some of the lay health promotion educators.

A second barrier identified by the site coordinators reflected concerns about whether or not the FHH materials were culturally appropriate for their communities. One coordinator reported that the FHH intervention offended some older members of the community because discussions of death and illness were taboo.

“But for a lot of [our community], when you ask them to do this, you’re stirring up old stuff. You’re asking them to think about family members and half of them are dead. So a lot of parents don’t want us asking their kids these questions. They say, ‘We don’t talk about that. We don’t bring it up. Why do they have to know that?’ There’s a whole group of [community members] who don’t come near us because they know we ask these questions. It’s not a benign process. We had to be kind of careful.”

Another coordinator discussed how some members of the community equated disease with divine justice and that information about FHH and chronic disease offended their religious beliefs. Recognizing these unique cultural barriers, the site coordinators reported that they tried to focus on segments of the community who would be more receptive to the topic. For example, the HHC included training on FHH collection in its prenatal case management program, on the assumption that expectant mothers would be more motivated to learn about disease risk and prevention if they believed it would benefit their child’s health.

A third and final barrier was the organizational capacity of each organization. While all of the site coordinators expressed interest in continuing to promote FHH, several of them were skeptical about their organization’s ability to sustain it. Previous work has shown that primary care providers can use FHH in a clinical encounter to tailor health promotion or disease screening for a patient [1,16,17,19,21]. In our pilot study, however, even those organizations that provide clinical services, such as the CHC and HHC, did not attempt to integrate it into these services—they instead built the FHH program into auxiliary programming using volunteers and community health workers. Coordinators believed that including it in routine clinical care would be prohibitively challenging because of the physicians’ large caseloads and limited time. In practice, however, this may mean that patients would collect FHH information but not necessarily share it with their provider and thus benefit from personalized healthcare. It also means that FHH becomes just one of many several health promotion programs the organization could potentially offer in the community, and makes the continuation of FHH dependent on funding or the interest and availability of staff members and volunteers providing those services. Some site coordinators stated that it would be difficult to replicate the intervention without ongoing and regular training, funds, and materials from the state health agency. They hoped that the state would continue its partnership and continue to make funding available for FHH promotion. One coordinator even hoped that the state would continue to assist community organizations in helping their underserved populations locate genetic services or find a way to pay for genetic testing that may be indicated by their FHH.

“I hope [the state agency] will move ahead to help do genetic testing. We want to be sure that our community understands the importance of genetically knowing who you are as well as knowing your family history. You can know your family history on multiple levels. So we’ll do the family history with or without the state but the next step for the state would be to say, ‘Hey let’s test a certain number of people and see what we can find out.’”

### 3.2. Outcome Evaluation

#### 3.2.1. Post-Test Participant Surveys

Three of the four community health organizations used the participant surveys in their interventions (N = 160). The CHC and the KHA disseminated and collected the surveys immediately after the program, and the HHC had an intern administer the survey via telephone. Table 3 shows the demographic makeup of the participants by organization. Only 138 of the 160 participants provided demographic information on the surveys.

**Table 3:** Demographic statistics of FHH participants (n = 138).

**Survey Questions**	**Community Health Center, Inc. (n = 65)**		**Hispanic Health Council (n = 20)**		**Khmer Health Advocates (n = 53)**		**Total (n = 138)**	
	N	%	N	%	N	%	N	%
**Gender**								
Female	44	67.7	20	100	34	64.2	98	71.0
Male	21	32.3	0	0	19	35.8	40	29.0
**Age**								
18 and Under	3	4.6	0	0	13	24.5	16	11.6
19–29	19	29.2	12	60.0	8	15.1	39	28.3
30–39	16	24.7	6	30.0	5	9.4	27	19.5
40–49	19	29.2	2	10.0	7	13.3	28	20.3
50+	8	12.3	0	0	20	37.7	28	20.3
**Latino/Hispanic**								
Yes	20	30.8	20	100	0	0	40	29.0
No	45	69.2	0	0	53	100	98	71.0
**Race**								
Asian	3	4.6	0	0	53	100	56	40.6
White	25	38.5	2	10.0	0	0	27	19.5
Black or African American	14	21.5	0	0	0	0	14	10.1
American Indian/Alaskan Native	1	1.5	1	5.0	0	0	2	1.5
Multi-racial	2	3.1	0	0	0	0	2	1.5
Other	1	1.5	0	0	0	0	1	0.7
Nonresponse	19	29.3	17	85.0	0	0	36	26.1

The majority of the sample identified as female (71.0%). This is unsurprising because women are more likely to seek healthcare and health education [8,47,60], and the HHC’s *Comadrona*/Healthy Start Program caters solely to women. Moreover, prior research has shown that women demonstrate greater levels of interest and knowledge in the topic of FHH than men [8,61,62]. Table 3 also shows that the sample was slightly younger than the state’s overall population (Connecticut’s median age is 40.2) and showed a higher proportion of nonwhite individuals (77.9% of Connecticut’s population identify as white) [41]. In fact, the majority of the sample identified as Latino/Hispanic (only 13.9% of Connecticut’s population identify as Latino/Hispanic) and Asian (only 4.0% of Connecticut’s population identify as Asian) [41]. This is indicative of the HHC and KHA’s participant populations. However, these findings are also representative of the underserved populations commonly served by community health organizations, which predominantly serve non-elderly adults aged 18-64 and non-white individuals [60,63].

The majority of respondents (85.6%) had seen a medical professional within the past 12 months (see Table 4). Only 20 of 139 participants had not seen a medical professional; the majority of these individuals (14) were affiliated with the KHA. The table also shows how the participants were made aware of the FHH intervention. The CHC’s community advertisements and on-site outreach techniques attracted the majority of their participants. The HHC and KHA had integrated their interventions into their regular programming, so participants were made aware by their enrollment in the program by staff or community health workers.

**Table 4:** Descriptive statistics of FHH participants previous visit to a medical provider and knowledge of FHH event (n = 139).

**Survey Questions**	**Community Health Center, Inc. (n = 66)**		**Hispanic Health Council (n = 20)**		**Khmer Health Advocates (n = 53)**		**Total (n = 139)**	
	N	%	N	%	N	%	N	%
**Times participant saw provider in the past 12 months**								
Have not seen health provider	5	7.6	1	5.0	14	26.4	20	14.4
Once	20	30.3	2	10.0	24	45.3	46	33.1
Twice	12	18.2	4	20.0	6	11.3	22	15.8
Three Times	10	15.1	3	15.0	2	3.8	15	10.8
Four Times or More	19	28.8	10	50.0	7	13.2	36	25.9
**How participant was made aware of FHH intervention**								
Community Advertisement	32	48.5	0	0	0	0	32	23.0
Community Health Worker	0	0	0	0	21	39.6	21	15.1
Enrolled in Program	0	0	20	100	0	0	20	14.4
On-Site	15	22.7	0	0	0	0	15	10.8
Nurse	0	0	0	0	12	22.6	12	8.6
Family Member/Friend	10	15.2	0	0	1	1.9	11	7.9
Doctor	7	10.6	0	0	1	1.9	8	5.8
Other	2	3.0	0	0	18	34.0	20	14.4

Participants reported overwhelmingly positive perceptions of the FHH interventions (Table 2). The majority of participants agreed that is important to know their FHH (85.6%) and that knowing their FHH decreases chances of developing future health problems (73%). The majority of participants also agreed that the staff members who presented the information were helpful (92.1%) and that the materials were useful (84.9% of the CHC and KHA participants). There were some subtle differences across groups in their contact with a healthcare provider in the past year. The Hispanic Health Council was specifically targeting this intervention to expectant mothers, so it is not surprising that half of this group reported four or more visits with a provider in the past year (or that all 20 of them reported hearing about the program from the community health worker who enrolled them). A far smaller proportion of participants at the health center and from the Khmer Health Advocates reported having seen a healthcare provider in the past year, which is more typical for these populations.

Table 2 also illustrates that participants agreed to share their FHH with their relatives (75%) or with a provider (82.9%). Approximately two-thirds of the sample also agreed to participate in another event that discussed FHH (67.1%). Also, 95 percent of the HHC’s participants agreed that they learned something new about FHH as a direct result of the intervention.

#### 3.2.2. Results from the Site Coordinator Follow-up Interviews

Site coordinators were skeptical of some findings from the participant surveys. Some did not believe participants would utilize the CT-DPH FHH workbook. When discussing the workbook, one site coordinator stated:

“It’s overwhelming and overly complicated and unlikely to be read by many people. There’s a lot of pieces that are buried in there that won’t be seen because people just see it as overwhelming.”

This sentiment was shared by another site coordinator who argued that the workbook better served as an initiator of conversation:

“I think some of the [participants] that we saw, [the workbook] might not have been as useful to them as we would’ve hoped. I think it was more about the dialogue between the person giving the information and the person receiving it. It’s something for them to hold and look at while you’re talking but I think our [participants] learn a lot through conversation than they do just by handing them a pamphlet and reading it.”

One coordinator asserted that participants would unlikely provide low ratings for staff or the materials because of an agreeability bias. She further argued that she was surprised they found the materials helpful, based on her knowledge of the low literacy levels common among her patients and clients.

Some of the coordinators were also interested in determining why some participants still did not want to share their FHH with relatives or a provider. A few coordinators suggested adding open-ended questions to the survey, while others suggested conducting post-intervention focus groups.

## 4. Discussion

This project shows that state health departments can be important and effective champions in disseminating information about FHH collection to underserved populations. State health departments have strong partnerships with many different kinds of organizations that reach different sectors of a community. In this project, the CT-DPH Genomics Office worked with a local health department, a community health center, and two community-based organizations that serve ethnic and immigrant minorities, to teach them about the importance of knowing one’s FHH, sharing it with relatives and healthcare providers, and the potential of using FHH information to personalize chronic disease prevention initiatives. Collectively, these interventions reached more than 100,000 Connecticut residents, with 160 participants completing a post-intervention follow up survey. The overwhelming majority of participants who completed surveys agreed that it is important to know their FHH, that knowing their FHH can help them prevent future health problems, and that they are willing to share their FHH information with relatives and healthcare providers.

These findings are not surprising, given that the CT-DPH’s intervention was based on a well-validated FHH educational tool—the U.S. Surgeon General’s *My Family Health Portrait*. However, despite the positive findings documented in the quantitative post-intervention survey, the qualitative interviews and observations give reason to interpret these findings with some caution. We make five recommendations about implementing FHH training in underserved communities.

First, several site coordinators worried about the novelty and complexity of genomics and chronic disease, especially for the low-literacy patients and clients they served. The interviews also showed that site coordinators had fairly sophisticated understandings of the relationship between genetic and environmental exposures—they understood that although families share a genetic endowment, they also share environments and social behaviors. The site coordinators were doubtful, however, that the workbook and materials conveyed this complexity to their patients and clients. On a related point, site coordinators were skeptical that their participants would be able to discern the difference between the general chronic disease prevention messages they disseminate to all participants from messages targeting high-risk populations. Future research should more thoroughly evaluate existing FHH curricula to assess how low-literacy or non-English speaking participants interpret the underlying cognates.

Second, site coordinators were concerned about whether their participants and clients would have access to healthcare services that would allow them to act on FHH knowledge to improve their health. This stemmed partly from a recognition that health care visits are very short and that busy PCPs may not have adequate time to consider FHH and tailor preventive care or screening services. It also, however, reflected beliefs that their patients may not have adequate insurance coverage that would cover genetic counseling and/or genetic testing. The Affordable Care Act, through the Essential Health Benefits element, requires insurers to cover counseling and testing for genetic tests that have received an A or B rating from the U.S. Preventive Services Task Force, but as of this writing, the only genetic test that has met this threshold of evidence is screening high-risk women for BRCA variants [64,65]. Some site coordinators feared that patients may collect FHH and discover that they are at high risk for other conditions (hypercholesterolemia, hereditary colorectal cancer), but that they may not have adequate coverage for genetic counseling and testing. Moreover, they worried that this might cause needless anxiety for their patients and clients. In this pilot study, the site coordinators avoided this problem by downplaying the importance of genetic counseling and genetic testing, but in the future, it would be good to train site coordinators about resources that may help patients defray the costs of genetic services. In any case, efforts to increase public awareness about the importance of knowing one’s family history does not mitigate the structural or clinical barriers in using that knowledge to guide decision making about risk stratification and follow up care for the patient.

Third, the state health agency very wisely allowed community-based organizations to tailor the intervention so that it fit with their previous and ongoing programming. This was a major incentive to the four groups that participated in this pilot program. However, this resulted in wide variation across community sites, and will make it difficult to determine whether the program overall met its goal of promoting FHH to underserved communities throughout the state. For example, while addressing FHH messages to the public via mass media may be effective in raising public awareness, the integration of the FHH message with a campaign about a seasonal public health threat such as influenza (as the local health department did in the holiday parade) may have been somewhat confusing. In the future, state health agency staff should perhaps monitor implementation plans more closely, to ensure that the FHH program is integrated logically and thematically with other chronic disease prevention programs.

Fourth, our pilot study provides some evidence that existing FHH materials may not be tailored sufficiently for culturally-specific populations. This concern was raised mainly by the KHA and the HHC, both of which said that the patients and clients they serve are often hesitant to discuss death and dying because of their migration history or because of religious beliefs. We had sought (but could not locate) a Khmer-language version of the FHH and chronic disease educational materials before launching this project. And while the Genetic Alliance project drew upon the expertise of patient advocates and oral historians, the program resulted in a single, standardized version of the *Does It Run In The Family*? FHH toolkit. While some of their community groups translated into additional languages or tailored the materials by adding English-language case studies of patients in Hmong communities, their toolkit was fairly generic and did not address all of the more subtle cultural aspects of health communication in diverse communities. There is a clear need for FHH educational materials in languages other than English and Spanish, and future research should be undertaken to identify ways to make those materials relevant to more culturally diverse groups.

Fifth, although all of the site coordinators had positive experiences with the FHH program, it is not clear that all of them will be able to sustain it as part of their regular programming. This was especially true for groups that relied upon volunteers or temporary health educators. As noted, those programs often rely on external funding, compete with other health promotion priorities (e.g., smoking cessation, nutrition and exercise), or depend on the interest and energy of a current crop of volunteers. On a related point, one of the groups in this pilot project was disappointed to learn that their plan of having youth educators train their elderly relatives would not work because of language barriers. In the future, state health agency staff may need to screen community-based organizations before implementation, to assess their capacity to reach the targeted populations and to integrate FHH collection over a longer term. If state health agency staff determine that reaching a particular underserved community with messages about FHH collection is especially important, they may need to make mini-grants or institutional support routinely available to support such programming.

There were some limitations to our evaluation methods. In an effort to minimize the administrative burden on site coordinators, we did not ask them to administer pre-intervention surveys to assess what patients or clients already knew about FHH and chronic disease. A baseline measure of participant knowledge, attitudes, and beliefs would have allowed us to determine if the FHH materials truly influenced participant knowledge. We also did not attempt to follow up with participants to learn whether they really did share FHH information with relatives or providers. Previous work in this area has shown that the practice of sharing FHH information with relatives and providers increases overall levels of communication and trust, makes nonparticipant relatives more willing to discuss health issues with their providers, and increases overall knowledge of risk factors associated with chronic diseases within the family [34,66,67], but those studies were done in a general population sample, and it is not clear whether they would be generalizable to the low-income and minority populations reached in this pilot program. More work is needed to determine whether a FHH educational program such as this one truly influences participant behavior around collecting and sharing FHH data. Finally, the demographic data on our participants show that the pilot program was somewhat successful at reaching underserved communities, especially ethnic and immigrant minorities, but women and younger adults were overrepresented in our population, relative to Connecticut’s population at large. More intensive efforts are needed to reach older patients and men with messages about FHH collection and chronic disease.

## 5. Conclusions

This pilot project shows how a state health department can be an important and effective champion in educating people about the links between FHH information and chronic disease prevention. It is especially noteworthy that the site coordinators found the topic of FHH and chronic disease prevention compatible with their existing health promotion programming, and that they appreciated the role of the CT-DPH Genomics Office’s program coordinator in providing current and reliable information about FHH. This shows the potential for state health agency staff to serve as an “honest broker” when it comes to introducing community partners to information about novel health promotion practices, including genomics and chronic disease prevention [68].

This project also shows how effective state health agencies can be in identifying and addressing unrecognized disparities in their states. The CT-DPH Genomics Office staff analyzed BRFSS data and recognized that some groups in their state were especially unlikely to benefit from a fairly simple risk assessment tool that could help to personalize health promotion. They developed this program to identify community-based organizations that were especially well situated to reach those groups and provided modest grants and technical support to assist them in this goal. This shows how state health agencies and community partners can, with modest investments of time and money, work to mitigate health disparities. Although more work is needed to determine how best to reach groups still unaddressed by projects such as these, and although public education and awareness campaigns such as these do not address the structural or clinical barriers in actually implementing FHH information to inform risk stratification and guide patient care, this pilot project outlines a model program that could be implemented in other states.
